# Association of general and abdominal adiposity with postural changes in systolic blood pressure: results from the NAKO pretest and MetScan studies

**DOI:** 10.1038/s41440-022-01029-5

**Published:** 2022-09-30

**Authors:** Ilais Moreno Velásquez, Lina Jaeschke, Astrid Steinbrecher, Heiner Boeing, Thomas Keil, Jürgen Janke, Tobias Pischon

**Affiliations:** 1 Max-Delbrück-Center for Molecular Medicine in the Helmholtz Association (MDC), Molecular Epidemiology Research Group, Berlin, Germany; 2 Department of Epidemiology (closed), German Institute of Human Nutrition Potsdam-Rehbruecke (DIfE), Nuthetal, Germany; 3 Institute of Social Medicine, Epidemiology and Health Economics, Charité—Universitätsmedizin Berlin, Berlin, Germany; 4 Institute of Clinical Epidemiology and Biometry, University of Würzburg, Würzburg, Germany; 5 State Institute of Health, Bavarian Health and Food Safety Authority, Bad Kissingen, Germany; 6 Max-Delbrück-Center for Molecular Medicine in the Helmholtz Association (MDC), Biobank Technology Platform, Berlin, Germany; 7 Berlin Institute of Health (BIH) at Charité—Universitätsmedizin Berlin, Core Facility Biobank Berlin, Berlin, Germany; 8 Charité—Universitätsmedizin Berlin, Corporate Member of Freie Universität Berlin and Humboldt-Universität zu Berlin, Berlin, Germany; 9 German Center for Cardiovascular Research (DZHK), Partner Site Berlin, Berlin, Germany

**Keywords:** Body mass index, Orthostatic blood pressure, Postural changes in systolic blood pressure, Waist circumference

## Abstract

The association between anthropometric measurements and postural changes in systolic blood pressure (SBP) has not been frequently reported. This study aimed to investigate the association of body mass index (BMI) and waist circumference (WC) with postural changes in SBP in two German cross-sectional studies. Data were derived from 506 participants of the population-based German National Cohort (NAKO) pretest and from 511 participants of the convenience sample-based MetScan studies. Linear regression models were used to estimate the association between BMI and WC with the difference between standing and sitting SBP (dSBP). Odds ratios (ORs) for an increase (dSBP > 10 mmHg) or decrease (dSBP ≤ −10 mmHg) in dSBP were calculated using logistic regression. The results were pooled by meta-analysis using an inverse variance model. In pooled analysis, a 5 kg/m^2^ higher BMI was associated with a 1.46 mmHg (95% confidence interval (CI) 0.98–1.94) higher dSBP, while a 5 cm higher WC was associated with a 0.51 mmHg (95% CI 0.32–0.69) higher dSBP. BMI or WC were associated with a higher odds of an increase in dSBP (adjusted OR, 1.71; 95% CI 1.36–2.14 per 5 kg/m^2^ higher BMI and 1.22; 95% CI 1.05–1.40 per 5 cm higher WC) but with a reduced odds of a decline in dSBP (adjusted OR, 0.67; 95% CI 0.44–1.00 per 5 kg/m^2^ higher BMI and 0.84; 95% CI 0.72–0.99 per 5 cm higher WC). The associations between WC and dSBP were no longer statistically significant after BMI adjustments. In conclusion, higher BMI and higher WC were associated with higher postural increases in SBP; however, WC was not related to postural changes in SBP once adjusted for BMI.

## Abstract (web summary)

Both, body mass index (BMI) and waist circumference (WC) were positively associated with postural dSBP, a measure of cardiovascular reactivity, in two independent populations from Germany. Further, WC was no longer significantly related to postural dSBP once BMI was accounted for.

## Graphical abstract

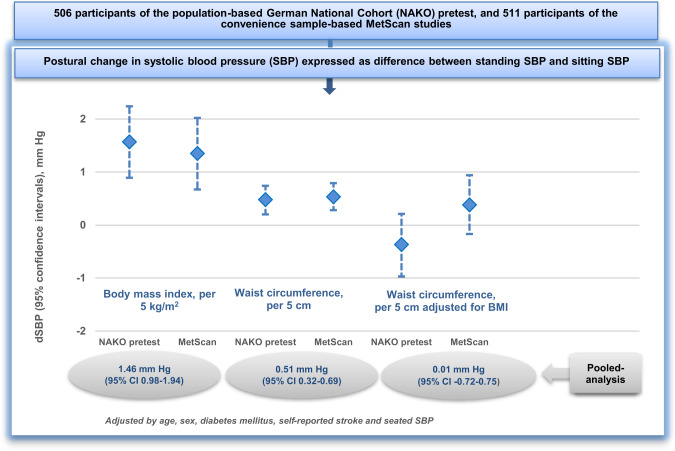

## Introduction

Short-term cardiovascular responses after orthostatic stress differ considerably among individuals and involve complex interactions between baroreceptor-mediated mechanisms and the autonomic nervous system [1]. Several population-based studies have reported orthostatic hypotension (OH) [defined as a systolic blood pressure (SBP)/diastolic blood pressure (DBP) decrease of at least 20/10 mmHg within 3 min of standing] [2] as a predictor for various cardiovascular disease (CVD) outcomes and all-cause mortality [3–6]. In contrast, the number of studies that examine postural blood pressure (BP) response as a quantitative trait is limited, and thus, there are questions open as to whether subtler changes (increases or decreases) in postural BP response are related to disease risk.

Findings from the Atherosclerosis Risk in Communities (ARIC) study, a cohort of middle-aged participants, suggest that both increases and decreases in the response of SBP to a change in body position may confer a higher risk of incident hypertension[7]. However, after adjustment for seated SBP, this association was only observed in the group with a decrease in SBP. Moreover, while there was a U-shaped association between postural changes in SBP and incidence of lacunar stroke, only negative changes in SBP were associated with increased incidence of thrombotic and cardiometabolic stroke [8]. These findings suggest that different biological mechanisms may underlie the two responses in BP upon standing. Likewise, a study in a community of elderly men reported a linear dose‒response relationship between postural changes in SBP and all-cause mortality [9].

In parallel, relatively little is known about the predisposing factors for subtle postural changes in SBP, regardless of their direction. This information could provide clues of potential pathophysiological relevance for BP control. Increasing levels of general and abdominal adiposity are important risk factors for incident stroke and hypertension [10, 11]. A higher body mass index (BMI) was reported in those with an increase in SBP upon standing in the ARIC study [7, 12], whereas a higher BMI was associated with a lower likelihood of OH in a community of mid- to late-life adults [13]. However, the association between anthropometric measurements and postural changes in SBP has not been frequently reported.

Thus, we aimed to investigate whether BMI and waist circumference (WC) were associated with postural changes in SBP and subtler changes in SBP in two cross-sectional studies on adult populations in Germany. We also tested the hypothesis that associations would be stronger among those with higher levels of seated SBP and DBP at baseline.

## Materials and methods

### Pretest of the German National Cohort (NAKO)

NAKO is an ongoing population-based study initiated in March 2014 that includes 205,000 participants recruited in 18 study centers across Germany, which is designed to investigate a broad range of potential causes of major disease groups [14]. To implement standardized study protocols and build infrastructure in the NAKO, two pretests were conducted during the period of May 2012 to April 2013 [15]. An invitation was sent by mail to eligible subjects (20- to 69-year-old residents in the catchment area of the municipal registries covered by the respective study center) informing them about the study. Approximately 200 participants were randomly recruited per study center. In the present study, we utilized data collected from participants enrolled in three study centers in the Berlin-Brandenburg cluster area (Max Delbrück Center for Molecular Medicine in the Helmholtz Association (MDC), Charité—Universitätsmedizin Berlin and the German Institute of Human Nutrition). In addition to computer-assisted personal interviews, health screening examinations and blood sampling under fasting conditions, these centers implemented an extended BP protocol with the aim of studying its feasibility in terms of acceptance, duration and technical-methodological issues. The protocol is an adapted version from the one utilized in the ARIC study [16]. Participants who reported a history of dizziness or syncope in response to a change in body position were excluded from the study protocol. In total, 653 individuals participated in the NAKO pretest 2, out of which 644 subjects underwent the extended BP protocol (398 women and 246 men) (Supplementary Fig. 1). Initially, version 1 (*n* = 122) of the extended BP protocol included shorter time intervals between standing measurements; however, due to feasibility reasons, a longer time interval between measurements was applied in versions 2 and 3. Thus, this analysis is based upon data obtained from 522 study participants from version 2/3 of the extended BP protocol. In addition, 28 participants out of the total sample underwent repeated measurements within 10 days for reliability assessment.

### The Metabolic Syndrome and Body Scan (MetScan) study

The cross-sectional MetScan study was established to investigate whether metabolic syndrome and its parameters can be better predicted using a 3D body surface scanner than using traditional recording methods [17, 18]. Between February 2016 and June 2017, eligible participants aged 18–79 years were recruited as a convenience sample by the MDC using a standardized recruitment protocol. Participants were asked to fill out a questionnaire to assess self-reported information on lifestyle and socioeconomic factors, medication intake and past medical history, as well as to undergo a physical examination that included the extended BP protocol and blood sampling. In total, 516 men and women agreed to be enrolled in the study (Supplementary Fig. 1). In agreement with the NAKO pretest, participants who reported a history of dizziness/syncope by postural changes were excluded from the BP protocol. Furthermore, 20 study participants out of the total sample underwent repeated BP measurements within 34 days for reliability assessment.

### Anthropometric measures

Measurements were performed manually by trained personnel according to World Health Organization (WHO) guidelines. Height (cm) was assessed using a Seca 285 stadiometer (Hamburg, Germany) or an equivalent model, weight (kg) was measured using a Seca mBCA-515 bioelectrical impedance analysis device (Hamburg, Germany) or an equivalent model, and WC (cm) was measured using Seca 201 tape (Hamburg, Germany) [15, 18]. BMI was calculated as body weight divided by the square of body height (kg/m^2^).

### Measurement of postural dSBP and outcome ascertainment

SBP and DBP (mmHg) were measured three times in the sitting position after 5 min of rest and recorded at 2-min intervals using an Omron Hem-705IT device. Sitting BP was calculated as the mean BP levels of the second and third measurements [19]. Thereafter, participants were asked to stand up, and the first standing BP measurement was taken. In the NAKO pretest, five measurements were recorded every 45 s (version 2/3) while the individual was standing. In methodological analyses, we observed that the fifth measurement did not provide significant information when classifying participants into different BP groups (agreement between inclusion and exclusion of the fifth measurement, as assessed using kappa coefficient was 0.86) [20], and therefore, the fifth measurement was omitted. In MetScan, the measurements were recorded using version 2/3 of the NAKO pretest, with the exception that the fifth standing measurement was not assessed.

To assess the reliability of standing BP based on the 28 and 20 participants in the NAKO pretest and MetScan, respectively, we calculated the intraclass correlation coefficient (ICC) and 95% confidence intervals (CIs) by analysis of variance by dividing the between-subject variance by the total variance (sum of between- and within-subject variances) [21]. Reliability for the first measurement was generally lower than that for the following measurements. Therefore, and since BP stabilization usually occurs during the first 30 s after standing [1], standing BP was defined as the average of the second to fourth measurement on standing. Based on this definition, the reliability of standing SBP was good (ICC: 0.70, 95% CI 0.45–0.85 in the NAKO pretest within 10 days of repeated measurement and 0.86, 95% CI 0.70–0.94 in MetScan within 34 days of repeated measurement, Supplementary Table 1).

Postural changes in SBP were expressed as the difference between standing SBP and sitting SBP (dSBP). In addition, we categorized postural dSBP into three groups using the following cutoff points: dSBP ≤ −10 mmHg (*decrease* in BP upon standing), dSBP >10 mmHg (increase in BP upon standing) and a range in postural dSBP between > −10 to ≤10 mmHg (stable BP upon standing). These categories are similar to those reported previously in the ARIC population [8].

### Statistical analysis

Out of the 522 study participants in the NAKO pretest, we further excluded 16 individuals with missing information on BMI, WC and BP measurements that precluded the calculation of dSBP, resulting in 506 participants (190 men, 316 women) finally being included in the analysis. In the MetScan study, 5 participants were excluded due to incomplete data on BP measurements. Thus, 511 subjects (187 men, 324 women) were included in the analysis.

Binary values are expressed as percentages, while quantitative variables are expressed as medians and interquartile ranges (IQRs). To optimize statistical power, the missing values for the following self-reported CVD risk variables were categorized as nonexposed: diabetes mellitus (DM) (*n* = 94, *n* = 9), stroke (*n* = 100, *n* = 9), current smokers (*n* = 106, *n* = 13), hypertension (*n* = 75, *n* = 14), and previous myocardial infarction (MI) (*n* = 78, *n* = 10) in the NAKO pretest and MetScan, respectively.

Linear regression analysis was used to estimate beta (β) coefficients and 95% CIs for the association of continuous BMI and WC with dSBP. The influence of potential confounders was addressed by adjusting for age (continuous), sex (male/female), DM (self-reported DM or fasting plasma glucose level >7 mmol/l or glycated hemoglobin level ≥48 mmol/mol), self-reported stroke (yes/no) and seated SBP (continuous). In further models, we examined the association of WC with dSBP with additional adjustment for BMI to assess to what extent WC was related to dSBP beyond what could be statistically accounted for by BMI. In an alternative approach, to avoid collinearity, we also examined the multivariable-adjusted association of BMI-adjusted residuals of WC with dSBP but found almost identical results. In a sensitivity analysis, we excluded participants with missing data on these covariates and repeated all analyses (participants included *n* = 405 in the NAKO pretest and *n* = 501 in MetScan).

Other variables evaluated as potential confounding factors were smoking, antihypertensive medication and seasonality (i.e., spring, summer, fall, winter).

To examine the association between BMI, WC, and postural increases/decreases in SBP upon standing, odds ratios (ORs) with 95% CIs were calculated by applying unconditional logistic regression models. BMI and WC were evaluated as continuous and categorical exposures using standard definitions [22]. BMI (kg/m^2^) categories were defined according to the WHO definition as obesity (≥30), overweight (≥25 to <30) and the reference group normal weight (<25), whereas WC (cm) was dichotomized as ≥94 for men, ≥80 for women (high WC) and <94 for men, <80 for women (normal WC, reference group). We adjusted the models for the aforementioned confounders.

In addition, a meta-analysis of the results was performed using an inverse variance model and random effects model, expressed as pooled estimated effects (β, ORs) and 95% CIs.

We tested for sex differences by including statistical interaction terms with sex and both exposures in the linear models. Furthermore, we assessed the statistical interaction for age (60 years as a cutoff), self-reported stroke and DM. To evaluate whether BP could modify the association between anthropometric measurements and dSBP upon standing, we conducted statistical interactions and stratified analyses using 140/90 mmHg as a cutoff. Additional adjustments were performed for antihypertensive drug therapy, assessed according to the Anatomical Therapeutic Chemical classification codes C01-CO4 and C06-C09.

Calculations were performed using SAS v.7.1 and RevMan v.5.4.1 programs.

## Results

Table 1 summarizes the distribution of selected baseline characteristics by study population. Overall, baseline characteristics did not differ across studies, with the exception of self-reported stroke, smoking, and monthly household income <1500 euros, which presented a higher prevalence in the NAKO pretest, whereas the proportion of participants with DM was higher in MetScan. The average postural response in dSBP (mmHg) was similar across the NAKO pretest (mean 1.7, standard deviation (SD) 7.0, range −20.8–29.5, median 1.7) and MetScan (mean 1.9, SD 6.6, range −21.7–27.5, median 2.0). The proportions of study participants who had increases in postural SBP upon standing were 9.9% in the NAKO pretest and 9.2% in MetScan. The proportions of participants who had decreases were 4.4% and 3.9%, respectively. Postural dSBP did not differ across sexes in either study. Orthostatic SBP changes presented higher variability than orthostatic DBP changes (NAKO pretest: SD for DBP change: 4.3 mmHg, SD for SBP change: 7.0 mmHg) (MetScan study: SD for DBP change: 3.9 mmHg, SD for SBP change: 6.7 mmHg).

**Table 1:** Distribution of baseline characteristics of the study participants of the population-based NAKO pretest and the MetScan studies

	NAKO-pretest study			MetScan study		
	All *n* = 506	Men *n* = 190	Women *n* = 316	All *n* = 511	Men *n* = 187	Women *n* = 324
Age (years)	52 (43–62)	54 (43–63)	52 (42–60)	52 (36–65)	55 (40–68)	52 (34–63)
Monthly household income, %						
<1500 €	17.8	14.7	19.8	11.4	6.9	14.0
1500–2999 €	41.7	42.9	40.9	43.8	38.4	47.0
3000–5999 €	36.9	39.6	35.4	39.2	45.9	35.1
≥6000 €	3.4	2.8	3.8	5.7	8.7	3.9
Current smokers^a^, %	19.4	25.3	15.8	9.6	8.6	10.2
Diabetes Mellitus, %	6.1	6.3	6.0	7.4	8.0	7.1
Hypertension, %	27.7	33.7	24.1	27.8	32.1	25.3
Stroke, %	2.2	3.2	1.6	1.6	0.5	2.2
Previous MI, %	1.2	2.6	0.3	1.4	1.6	1.2
BMI, kg/m²	25.3 (23.1–28.9)	26.9 (24.4–29.6)	24.4 (22.2–27.8)	25.4 (22.5–28.3)	25.9 (24.0–28.3)	24.5 (21.7–28.3)
WC, cm	88.5 (79.1–98.4)	96.0 (90.1–105.5)	83.6 (75.7–92.2)	88.0 (77.5–98.8)	95.5 (87.1–104.6)	83.1 (74.0–93.1)
Waist-hip ratio	0.9 (0.8–0.9)	1.0 (0.9–1.0)	0.8 (0.8–0.9)	0.9 (0.8–0.9)	0.9 (0.9–1.0)	0.8 (0.7–0.9)
Waist-height ratio	0.5 (0.5–0.6)	0.5 (0.5–0.6)	0.5 (0.5–0.6)	0.5 (0.4–0.6)	0.5 (0.5–0.6)	0.5 (0.4–0.6)
Total cholesterol, mg/dl	224.4 (193.3–247.5)	220.4 (189.5–247.5)	224.0 (193.3–247.5)	216.6 (185.6–243.6)	212.7 (185.6–239.7)	216.7 (185.6–247.5)
HDL-cholesterol, mg/dl	54.1 (46.4–69.9)	50.3 (42.5–58.0)	61.9 (50.3–73.5)	58.0 (50.3–69.6)	54.1 (46.4–58.0)	61.9 (54.1–73.5)
LDL-cholesterol, mg/dl	–	–	–	131.5 (108.3– 158.6)	135.3 (108.3– 162.4)	131.5 (108.3– 158.6)
HbA1c, mmol/mol	38.6 (35.3–40.8)	38.6 (36.4–40.8)	37.5 (35.3–40.8)	35.0 (32.0–38.0)	35.0 (32.0–38.0)	35.1 (32.0–38.6)
Antihypertensive therapy, %	22.7	30.0	18.4	23.8	27.3	21.9
SBP (mmHg)	124 (114–134)	129 (121–138)	119 (109–130)	121 (112–133)	129 (119–138)	116 (108–126)
DBP (mmHg)	77 (71–84)	80 (74–84)	75 (70–81)	76 (70–82)	79 (73–84)	74 (68–80)
Ambient temperature (°C)	23.3 (22.6–24.1)	23.3 (22.7–24.1)	23.3 (22.6–24.1)	22.0 (21.0–23.0)	22.0 (21.0–23.0)	22.0 (21.0–23.0)
Postural changes in SBP, mmHg	1.7 (−2.8–5.7)	1.7 (−3.0–5.7)	1.7 (−2.1–5.9)	2.0 (−2.0–6.0)	2.0 (−2.5–5.8)	2.0 (−1.6–6.1)
Postural change in SBP (mmHg) category, %						
Decline ≤ −10	4.4	4.2	4.4	3.9	2.7	4.6
Decline > −10 to ≤−5	11.5	11.1	11.7	9.8	10.2	9.6
Decline > -5 to Increase ≤5	54.6	55.3	54.1	55.6	58.8	53.7
Increase ≥5 to ≤10	19.8	20.5	19.3	21.5	20.8	21.9
Increase >10	9.9	8.9	10.4	9.2	7.5	10.2
Postural changes in DBP, mmHg	4.0 (1.4–6.3)	3.5 (1.1–1.5)	4.1 (1.5–6.3)	4.5 (2.0–7.5)	4.0 (1.5–6.8)	5.0 (2.4–7.6)
Postural change in DBP (in mmHg) category, %						
Decline ≤ −10	0.2	–	0.3	–	–	–
Decline > −10 to ≤0	15.8	16.3	15.5	12.1	12.3	12.0
Increase >0 to ≤10	76.3	77.4	75.6	77.8	81.8	75.6
Increase >10	7.7	6.3	8.5	9.9	5.9	12.3

Continuous variables expressed as median (IQR). Missing values: Monthly household income (*n* = 41, *n* = 54), total cholesterol (*n* = 7, *n* = 11), HbA1c (*n* = 106, *n* = 13), HDL-cholesterol (*n* = 106, *n* = 11), LDL-cholesterol (*n* = 11), in NAKO-pretest and MetScan, respectively

*CVD* cardiovascular diseases, *MI* myocardial infarction, *BMI* body mass index, *WC* waist circumference, *SBP* systolic blood pressure, *DBP* diastolic blood pressure, *HbA1c* glycated hemoglobin

^a^Smoker variable: NAKO-pretest: never, current, former-smokers. MetScan: never, current, former-smokers defined as having smoked until 3 months previous to the enrollment

The distribution of characteristics of the study populations was, in general, similar for those who did not have missing covariate information (Supplementary Table 2).

The multivariable association of BMI and WC with postural dSBP is shown in Table 2. A 5 kg/m^2^ higher BMI was associated with a 1.57 mmHg higher postural dSBP in the NAKO pretest (95% CI 0.89–2.24). Similarly, a 5 kg/m^2^ higher BMI was associated with a 1.35 mmHg higher dSBP in MetScan (95% CI 0.67–2.02) (fully adjusted models). The pooled estimate was 1.46 mmHg per 5 kg/m^2^ higher BMI (95% CI 0.98–1.94).

**Table 2:** Association of body mass index (per 5 kg/m^2^) and waist circumference (per 5 cm) with postural change in blood pressure (in mmHg)

	dSBP (95% CI)		
	NAKO-pretest study (*n* = 506)	MetScan study (*n* = 511)	Pooled estimates
BMI, per 5 kg/m^2^			
Crude	1.48 (0.85–2.10)	0.98 (0.36–1.60)	1.23 (0.74–1.72)
Model 1	1.52 (0.87–2.18)	1.04 (0.37–1.70)	1.29 (0.81–1.76)
Model 2	1.38 (0.71–2.05)	1.12 (0.44–1.80)	1.25 (0.77–1.73)
Model 3	1.57 (0.89–2.24)	1.35 (0.67–2.02)	1.46 (0.98–1.94)
WC, per 5 cm			
Crude	0.36 (0.14–0.58)	0.28 (0.08–0.48)	0.32 (0.17–0.46)
Model 1	0.48 (0.22–0.74)	0.41 (0.16–0.66)	0.44 (0.26–0.62)
Model 2	0.41 (0.14–0.67)	0.44 (0.18–0.69)	0.42 (0.24–0.60)
Model 3	0.48 (0.20–0.74)	0.53 (0.28–0.79)	0.51 (0.32–0.69)
Model 4 (adjusted for BMI)	−0.37 (−0.95–0.22)	0.38 (−0.17–0.94)	0.01 (−0.72–0.75)

Model 1: adjusted by age and sex. Model 2: Model 1 + adjusted by diabetes mellitus and self-reported stroke. Model 3: Model 2 + adjusted by seated SBP. Model 4: Model 3 + adjusted for BMI. Results were almost identical when we included BMI-adjusted WC residuals in the model

*BMI* body mass index, *WC* waist circumference, *SBP* systolic blood pressure

Similarly, a 5 cm higher WC was associated with a 0.48 mmHg higher postural dSBP in the NAKO pretest (95% CI 0.20–0.74) and with a 0.53 mmHg higher dSBP in MetScan (95% CI 0.28–0.79) (Model 3). The pooled estimate was 0.51 mmHg per 5 cm higher WC (95% CI 0.32–0.69). As expected, BMI and WC were highly correlated (Spearman’s correlation coefficient (*r*), 0.87; *p* < 0.0001 in the NAKO pretest and 0.87, *p* < 0.0001 in MetScan). When additionally adjusted for BMI, the association of WC with dSBP was no longer statistically significant (Table 2, Model 4). The results were almost identical when we used a model that included BMI and BMI-adjusted WC residuals (data not shown). Moreover, our models were unaltered after inclusion of antihypertensive therapy and smoking: a 5 kg/m^2^ higher BMI was associated with a 1.49 mmHg higher postural change in SBP (95% CI 0.80–2.17), and a 5 cm higher WC was associated with a 0.43 mmHg higher postural change in SBP (95% CI 0.16–0.70) (unadjusted for BMI) in the NAKO pretest. In the MetScan study, a 5 kg/m^2^ higher BMI was associated with a 1.36 mmHg higher postural change in SBP (95% CI 0.67–2.06), and a 5 cm higher WC was associated with a 0.53 mmHg higher postural change in SBP (95% CI 0.26–0.79). BMI-adjusted models remained unchanged for the association of WC with postural changes in SBP after further adjustments for these variables (NAKO pretest: *β* −0.42; 95% CI −1.00–0.16 and in MetScan: *β* 0.29; 95% CI −0.28–0.87). Likewise, the inclusion of seasonality into the models of BMI (per 5 kg/m^2^) (NAKO pretest *β* 1.45, 95% CI 0.77–2.13; MetScan *β* 1.37, 95% CI 0.68–2.04) and into the models of WC (per 5 cm) (NAKO pretest *β* 0.41, 95% CI 0.14–0.68; MetScan *β* 0.54, 95% CI 0.28–0.80) with postural changes in BP did not change the point estimates substantially. Similarly, our BMI-adjusted models remained unaltered for the association of WC with postural changes in SBP after further inclusion of seasonality (NAKO pretest *β* −0.46, 95% CI −1.05–0.12; MetScan *β* 0.39, 95% CI −0.16–0.95).

When we cross-classified participants based on BMI and WC, we found the largest increase in dSBP among obese participants with high WC (3.8 mmHg; IQR −0.7; 8.0 in the NAKO pretest and 2.9 mmHg; IQR −1.2; 8.3 in MetScan, compared to non-obese participants with normal WC or compared to non-obese participants with high WC, Table 3). Similarly, the proportion of individuals with an increase (>10 mmHg) in postural dSBP was higher in this group.

**Table 3:** Postural Changes in SBP (mmHg) in non-obese persons with normal or high waist circumference, and in obese persons with normal or high waist circumference

	Non-obese normal WC	Non-obese high WC	Obese normal WC	Obese high WC
NAKO-pretest study				
*n* (%)	204 (40.4)	194 (38.3)	0	108 (21.3)
Mean ± SD	0.8 ± 6.4	1.3 ± 6.7	–	4.1 ± 8.1
Median (IQR)	1.1 (−3.7–5.0)	1.3 (−3.2–5.5)	–	3.8 (−0.7–8.0)
Minimum	−19.5	−18.8	–	−20.8
Maximum	25.8	29.5	–	26.5
Decline ≤10 mmHg in SBP on standing, *n* (%)	12 (5.9)	6 (3.1)	0	4 (3.7)
Increase >10 mmHg in SBP on standing, *n* (%)	13 (6.4)	17 (8.8)	0	20 (18.5)
MetScan study				
*n* (%)	231 (45.2)	194 (38.0)	0	86 (16.8)
Mean ± SD	1.0 ± 5.4	2.5 ± 6.8	–	3.4 ± 8.7
Median (IQR)	1.2 (−2.7–5.2)	2.7 (−1.3–7.7)	–	2.9 (−1.2–8.3)
Minimum	−19.2	−21.7	–	−19.8
Maximum	14.2	19.7	–	27.5
Decline ≤10 mmHg in SBP on standing, *n* (%)	7 (3.0)	9 (4.6)	0	4 (4.7)
Increase >10 mmHg in SBP on standing, *n* (%)	6 (2.6)	25 (12.9)	0	16 (18.6)

Continuous variables expressed as median (interquartile range, IQR). Obesity: BMI ≥ 30 kg/m^2^. High WC: WC ≥ 94 cm in men, WC ≥ 80 cm in women. Normal WC: WC < 94 cm in men, WC < 80 cm in women

*WC* waist circumference, *SBP* systolic blood pressure, *BMI* body mass index

Table 4 presents the association of continuous and categorical anthropometric measurements with the likelihood of having (1) either a >10 mmHg increase or a ≤10 mmHg decrease in postural SBP; (2) a >10 mmHg increase in postural SBP; and (3) a ≤−10 mmHg decrease in postural SBP.

**Table 4:** Association between anthropometric measurements and postural changes in SBP, expressed as odds ratio (OR), 95% confidence intervals (CI)

	Odds ratio for the specified change in SBP upon standing		
	Increase or decrease in SBP	Increase in SBP (>10 mmHg in SBP on standing)	Decrease in SBP (≤−10 mmHg in SBP on standing)
**NAKO-pretest study (*n* = 506)**			
BMI, per 5 kg/m^2^	1.24 (0.95–1.61)	1.53 (1.14–2.05)	0.61 (0.35–1.06)
BMI categories			
Normal weight	1 (reference)	1 (reference)	1 (reference)
Overweight	0.97 (0.51–1.87)	1.46 (0.66–3.21)	0.50 (0.16–1.53)
Obese	1.66 (0.87–3.18)	2.76 (1.27–5.99)	0.50 (0.15–1.69)
WC, per 5 cm, not adjusted for BMI	1.04 (0.93–1.16)	1.13 (1.00–1.27)	0.82 (0.66–1.02)
WC, per 5 cm, adjusted for BMI	0.82 (0.64–1.05)	0.80 (0.60–1.08)	0.89 (0.59–1.36)
WC categories, not adjusted for BMI			
Normal WC	1 (reference)	1 (reference)	1 (reference)
High WC	0.94 (0.54–1.64)	1.41 (0.70–2.80)	0.45 (0.18–1.14)
WC categories, adjusted for BMI			
Normal WC	1 (reference)	1 (reference)	1 (reference)
High WC	0.63 (0.31–1.25)	0.77 (0.33–1.77)	0.67 (0.22–2.09)
**MetScan study (*n* = 511)**			
BMI, per 5 kg/m^2^	1.57 (1.18-2.07)	1.93 (1.42-2.63)	0.74 (0.40-1.34)
BMI categories			
Normal weight	1 (reference)	1 (reference)	1 (reference)
Overweight	2.90 (1.45–5.83)	6.76 (2.59–17.59)	0.51 (0.16–1.69)
Obese	3.87 (1.78–8.43)	9.71 (3.47–27.14)	0.51 (0.12–2.11)
WC, per 5 cm, not adjusted for BMI	1.19 (1.06–1.32)	1.31 (1.16–1.48)	0.87 (0.69–1.09)
WC, per 5 cm, adjusted for BMI	1.09 (0.86–1.40)	1.21 (0.91–1.60)	0.84 (0.53–1.34)
WC categories, not adjusted for BMI			
Normal WC	1 (reference)	1 (reference)	1 (reference)
High WC	3.26 (1.59–6.71)	6.69 (2.55–17.55)	0.68 (0.21–2.20)
WC categories, adjusted for BMI			
Normal WC	1 (reference)	1 (reference)	1 (reference)
High WC	2.32 (0.99–5.40)	3.93 (1.33–11.58)	1.00 (0.23–4.38)
**Pooled estimates**			
BMI, per 5 kg/m^2^	1.39 (1.10–1.75)	1.71 (1.36–2.14)	0.67 (0.44–1.00)
BMI categories			
Normal weight	1 (reference)	1 (reference)	1 (reference)
Overweight	1.67 (0.57–4.88)	3.06 (0.68–13.77)	0.50 (0.22–1.14)
Obese	2.47 (1.08–5.63)	4.94 (1.44–16.89)	0.51 (0.20–1.28)
WC, per 5 cm, not adjusted for BMI	1.11 (0.97–1.27)	1.22 (1.05–1.40)	0.84 (0.72–0.99)
WC, per 5 cm, adjusted for BMI	0.95 (0.71–1.26)	0.99 (0.66–1.48)	0.87 (0.64–1.19)
WC categories, not adjusted for BMI			
Normal WC	1 (reference)	1 (reference)	1 (reference)
High WC	1.71 (0.51–5.80)	2.95 (0.64–13.61)	0.53 (0.26–1.10)
WC categories, adjusted for BMI			
Normal WC	1 (reference)	1 (reference)	1 (reference)
High WC	1.18 (0.33–4.24)	1.67 (0.34–8.27)	0.78 (0.32–1.92)

BMI (kg/m^2^) categories were defined according to the WHO definition as normal weight (<25), overweight (≥25 to <30), or obese (≥30); WC (cm) was dichotomized as normal WC (<94 in men, <80 in women), or high WC (≥94 in men, ≥80 in women).

Model 1: adjusted by age, sex, diabetes, stroke and seated SBP

*BMI* body mass index, *WC* waist circumference, *SBP* systolic blood pressure

In MetScan, a 5 kg/m^2^ higher BMI was statistically significantly associated with a 1.57-fold OR of having a postural increase or decrease in SBP (95% CI 1.18–2.07), while a 5 cm higher WC was significantly associated with a 1.19-fold OR (95% CI 1.06–1.32) for an increase or a decrease. When compared to the normal weight study population, participants who were overweight had an OR of 2.90 (95% CI 1.45–5.83), and participants with obesity had a 3.87-fold OR (95% CI 1.78–8.43) of having an increase or a decrease in postural SBP; individuals with a high WC compared to those with a low WC had a 3.26-fold OR (95% CI 1.59–6.71).

A 5 kg/m^2^ higher BMI was associated with a 1.53-fold OR (95% CI 1.14–2.05) of having a postural increase in dSBP in the NAKO pretest and a 1.93-fold OR (95% CI 1.42–2.63) in MetScan. Compared to the normal weight BMI category, obesity was associated with a 2.76-fold (95% CI 1.27–5.99) OR of having an increment in postural SBP in the NAKO pretest and a 9.71-fold (95% CI 3.47–27.14) OR in MetScan. In MetScan, this association was also observed for overweight (OR 6.76, 95% CI 2.59–17.59), yet with a lower point estimate.

Similarly, a 5 cm higher WC was associated with a 1.13-fold (95% CI 1.00–1.27) OR of having an increment in postural dSBP in the NAKO pretest and a 1.31-fold (95% CI 1.16–1.48) OR in the MetScan. These associations were attenuated after adjustments for BMI. Compared to the low WC category, high WC was associated with an OR of 6.69 for an increment in dSBP in MetScan (95% CI 2.54–17.54) and remained statistically significant after adjustment for BMI.

In pooled analysis, BMI or WC were associated with higher odds of an increase in dSBP (OR, 1.71; 95% CI 1.36–2.14 per 5 kg/m^2^ higher BMI and 1.22; 95% CI 1.05–1.40 per 5 cm higher WC). No statistically significant association was observed between the anthropometric measurements and a decrease in dSBP in either of the studies, although the number of cases was low. However, in the pooled analysis, BMI or WC were associated with reduced odds of a decline in dSBP (OR, 0.67; 95% CI 0.44–1.00 per 5 kg/m^2^ higher BMI and 0.84; 95% CI 0.72–0.99 per 5 cm higher WC).

In the NAKO pretest, a 5 kg/m^2^ higher BMI was statistically significantly associated with a 3.30 mmHg (95% CI 1.92–4.68) higher postural dSBP in individuals with a sitting BP ≥ 140/90 mmHg (Supplementary Table 3). Similarly, a 5 cm higher WC was significantly associated with a 1.37 mmHg (95% CI 0.79–1.94) higher postural dSBP in individuals with a sitting BP ≥ 140/90 mmHg but not in those with a sitting BP < 140/90 mmHg. In MetScan, the interaction terms were not statistically significant, and we observed significant associations of anthropometric measures with postural dSBP in both groups. However, consistent with the NAKO pretest, the point estimates were lower among those with BP < 140/90 mmHg than among those with BP ≥ 140/90 mmHg. Similarly, as for the main analysis, all associations of WC with postural dSBP were attenuated after adjustments for BMI. Further adjustments of the association of BMI and postural dSBP by antihypertensive treatment among those with BP ≥ 140/90 had a minimal impact on the point estimates in the NAKO pretest (OR 3.30, 95% CI 1.89–4.71) and in MetScan (OR 2.07, 95% CI 0.42–3.72) (data not shown).

Neither the NAKO pretest nor MetScan showed evidence of a statistical interaction for age and anthropometric measurements: in the NAKO pretest, the *p* value for BMI × age 60 was 0.22 and *p* value for WC × age 60 was 0.23; in MetScan, the *p* value for BMI × age 60 was 0.50 and *p* value for WC × age 60 was 0.32. However, the point estimates for the association between anthropometric measurements and postural changes in SBP were higher in the population older than 60 years of age (Supplementary Table 5).

Likewise, the interaction terms for sex × BMI and sex × WC were non-statistically significant: in the NAKO pretest, the *p* value for BMI × sex was 0.84, and the *p* value for WC × sex was 0.21. In MetScan, the interaction terms were BMI × sex (*p* = 0.06) and WC × sex (*p* = 0.12).

In the NAKO pretest, the interaction terms for stroke × BMI (*p* = 0.0004) and stroke × WC (*p* = 0.002) were statistically significant, yet the few cases precluded us from performing stratified analyses. However, sensitivity analyses excluding study participants with a self-reported history of stroke showed that the point estimates were lower, although there was a similar direction of the effect (data not shown).

## Discussion/conclusion

The main finding of the current study was that both BMI and WC were positively associated with postural changes in SBP, a measure of cardiovascular reactivity, in two independent populations from Germany. Furthermore, WC was no longer significantly related to postural changes in SBP once BMI was accounted for (either by direct adjustment or by using BMI-adjusted WC residuals in the models). These data suggest that adiposity in general is associated with a larger increase in postural changes in SBP, whereas body fat distribution as assessed by WC is not additionally related to postural changes in SBP beyond BMI. Our results also suggest that sitting BP levels may modify these associations.

The present findings are in agreement with the ARIC study, in which those with postural increases in SBP had higher BMIs than those in the reference group, while decreases in SBP were not significantly related to BMI [7]. Our results indicated that participants with obesity and high WC had higher values of postural changes in SBP than the overall study population. Likewise, the proportion of those with an increase in SBP upon standing was higher in the study population with obesity and high WC. Both exposures analyzed as continuous variables had similar associations with our outcome of interest across studies. Furthermore, once adjusted for BMI, the association between WC and postural changes in SBP was no longer significant. These data suggest that adiposity in general plays a major role, whereas abdominal body shape does not add further information.

Observational studies assessing health-related correlates of subtle postural BP increases are scarce; nevertheless, there are some potential mechanisms that may explain our findings. Under physiological circumstances, SBP drops by 5–10 mmHg after changing position [23], and factors such as the autonomic nervous system, intravascular volume, duration of erect posture, postprandial state and temperature (ambient, indoor and clinical) may influence the homeostasis of BP [24, 25]. Adiposity appears to impair autonomic function, potentially explained by alterations in insulin- and leptin-mediated sympathetic nervous system activation and baroreceptor dysfunction [26–29]. Interestingly and in line with our pooled estimates, previous studies have reported inverse relationships between BMI and OH in both men and women and in participants with neurological conditions [13, 28, 30]. Furthermore, recent evidence suggests that baroreflex sensitivity is impaired in metabolic syndrome [31] and that there is a link between inflammatory pathways and autonomic dysfunction and atherothrombosis in OH [32]. In addition, postural changes in SBP and BMI appear to have a joint genetic regulation by one or more genes on Chr. 13q [33]. In this context, questions arise as to when subtle postural changes in SBP begin in the lifetime and whether subtle postural increases in SBP are an earlier measure of the disease process or an independently acting mechanism [34]. In fact, a mean postural increase in SBP of 15.2 mmHg (range 11.3–43.8 mmHg) was associated with incident hypertension in the ARIC population; however, the association did not remain statistically significant after adjustments for seated SBP [7]. In addition, the ARIC study showed that values of postural change in SBP lower than 20 mmHg conferred elevated hazard ratios for lacunar stroke [8]. Similarly, higher odds for silent cerebral infarction were also observed for values of postural SBP change lower than 20 mmHg in an older population from Japan [35]. In the context of our findings, 9% of the participants in both the NAKO pretest and the MetScan studies had an increase of more than 10 mmHg in SBP upon standing. This frequency increased up to 16.9% in the NAKO pretest and to 12.4% in the MetScan study when only the population older than 60 years of age was considered. Taken together, one may speculate that these groups of participants might be at an elevated risk of overt clinical cerebrovascular events. Nevertheless, our cross-sectional design limits us from drawing clinical interpretations. Therefore, further research in the field of orthostatic changes in BP (in particular increases) and their clinical validation are essential to elucidate prevalence, incidence, and independent prognostic significance in different populations.

In contrast to previous population-based studies in which median values of postural changes in SBP were near 0 mmHg [4, 7], our mean value of postural dSBP was between 1.7 and 2.0 mmHg. This discrepancy might be partly explained by our exclusion criteria, in which study participants with a history of dizziness or syncope by a change in posture were not included as part of the extended BP protocol. Therefore, the present findings should be interpreted in relation to a population that excludes people with symptomatic systolic OH. Nevertheless, our aim was to study subtler responses to postural changes in SBP rather than clinical OH. Notably, 0.4% and 0.8% of the NAKO pretest and MetScan study participants, respectively, included in this study had asymptomatic postural dSBP ≥20 mmHg (data not shown). In addition, sex differences in BP regulation have been previously reported [12, 36], and there is evidence of sex differences in BP hemodynamics in overweight and obese populations [37]. However, we found similar responses to systolic postural changes among men and women. Consistent with the ARIC study [8], we observed less variability in orthostatic DBP changes than in postural SBP changes. Consequently, we focused our analysis on postural changes in SBP. Moreover, in a complementary analysis, we did not find associations between anthropometric measurements and dDBP, except for BMI in the NAKO pretest: a 5 kg/m^2^ higher BMI was associated with a 0.64 mmHg higher postural dDBP (95% CI 0.21–1.07) (data not shown).

We further evaluated the modification of the main effect by baseline, seated SBP, and our associations were stronger among those with higher levels of seated SBP. In hypertensive populations, baroreceptor responsiveness is impaired, possibly through different mechanisms [38]. It has been suggested that antihypertensive therapy may have an impact on postural changes in SBP [3, 39]; nonetheless, our findings were independent of antihypertensive treatment use.

One of the issues that emerges from our study is the moderate to good reliability in SBP measurement assessment. The superior reliability in MetScan, despite longer days in between measurements, might be explained by the number of trained nurses who performed the repeated measurements (4 nurses) compared to the NAKO pretest, in which more trained nurses participated in the pretest and might have influenced the intervariability. Symptomatic postural changes in BP are measured using a head-up tilt test; however, this test is not always available and warrants interpretation by a well-trained expert [39]. Thus, a corollary of this observation is that the protocol utilized here may be feasible in large population-based epidemiological studies. This is also underlined by the fact that the associations we observed between changes in SBP and BMI and WC were remarkably similar across the NAKO pretest and MetScan. In fact, in the fully adjusted models, a change of one SD in BMI was associated with a 0.21 SD and 0.19 SD increase in postural changes in BP in the NAKO pretest and MetScan studies, respectively. Similarly, a change of one SD in WC was associated with a 0.19 SD (NAKO pretest) and 0.23 SD (MetScan) increase in postural changes in BP, which were no longer statistically significant after adjustment for BMI (data not shown).

Furthermore, both supine-to-standing measurements and sitting-to-standing measurements are utilized in research settings. The ARIC study defined changes in SBP as the difference between standing and supine SBP, whereas in our study we used sitting instead of supine SBP. It has been suggested that the decrease in BP that occurs from transitioning from a supine to a standing position may be greater than that which occurs when changing position from sitting to standing [40]. If this is the case, then it is possible that our postural decreases in SBP were underestimated, as our study would have misclassified individuals who truly had postural decreases in SBP as not having them.

The strengths of this study include the standardized procedures used for postural BP measurements under similar indoor temperatures, the assessment of reliability of measurements and the replication of the findings in an independent population using continuous and categorical outcomes.

However, several limitations should be mentioned. First, due to the design, cross-sectional studies do not allow us to examine temporal relationships. Second, while we included known risk factors for OH as covariates, residual or unmeasured confounding (e.g., genetic predisposition [41], other neurological diseases [42]) needs to be considered when interpreting the results. Moreover, given that the covariates considered were all self-reported data, any potential misclassification of confounders should be taken into consideration. Nevertheless, the results were consistent when we analyzed confounding variables, taking missing data into account. Third, we cannot rule out that dSBP might be overestimated, as we excluded participants with a history of postural dizziness/syncope. Fourth, the MetScan study population was drawn as a convenience sample, and self-selection bias is likely with this approach. However, the characteristics were similar to those observed in the population-based sample from the NAKO pretest. Fifth, this study might have been underpowered to detect associations examining decreases in dSBP, and thus, the models should be interpreted with caution. Moreover, because it has been suggested that the assessment of DBP may be less reliable [34], we did not analyze postural changes in DBP (DBP usually increases by 5–10 mmHg upon standing due to peripheral vasoconstriction and reduction in cardiac stroke). Finally, one may argue that a considerable number of participants (*n* = 117) were excluded from the NAKO pretest due to the application of version 1 of the BP protocol. While we did not see differences in median values for BMI, WC or the prevalence of DM and stroke among those excluded, postural dSBP was slightly higher (Supplementary Table 4). Nevertheless, we were able to replicate the results in an independent population.

In conclusion, general adiposity was associated with postural changes in SBP in two independent populations, and this relationship was potentially driven by increases in postural SBP. Abdominal adiposity was not significantly related to changes in SBP once general adiposity was considered. Our results further suggest that seated BP levels seemed to modify this association.

Although the clinical implication of subtler postural changes in SBP is still unclear, our findings highlight the current knowledge gap in the epidemiology of orthostatic SBP dysregulation and the associated risk factors. Taken together, the mechanisms to maintain a normal BP postural response by adiposity may have implications for future research strategies. From a broader perspective, prospective studies that assess whether subtle increases in postural BP are associated with cardiovascular outcomes in different populations are of interest.

## Supplementary information

Supplementary Information

## Data Availability

The data that support the findings of this study are available from the authors, but restrictions apply to the availability of these data, which were used under license for the current study and are not publicly available. Interested researchers (who meet criteria for access to confidential data) may contact the corresponding author of our manuscript for access to the datasets generated and/or analyzed during the current study.
