# Supplementary material for: Association of general and abdominal adiposity with postural changes in systolic blood pressure: results from the NAKO pretest and MetScan studies
Source: Hypertens Res. 2022 Sep 30;45(12):1964–76. doi: 10.1038/s41440-022-01029-5 (PMC9659486; doi:10.1038/s41440-022-01029-5)
Supplement: Supplementary file 1 — Supplementary Information [file 41440_2022_1029_MOESM1_ESM.docx]

**Supplementary Figure 1. Flowchart of the study population in the NAKO-pretest (A) and MetScan (B) studies**

1. **NAKO-pretest**

**n= 653** study participants

(Berlin-Brandenburg cluster area)

**n= 644** participants

underwent the extended BP measurement protocol

n=9 participants excluded with history of dizziness or syncope in response to a change in body position

**n= 522** participants

(version 2-3 of the BP measurement protocol)

n=122 excluded

(version 1 of the BP measurement protocol)

**n= 28** participants

repeated measurements within 10 days for reliability assessment

**n= 506** participants

(190 men, 316 women)

n=16 excluded with

missing data on BMI, WC and/or BP measurements

1. **MetScan**

**n= 516** study participants

(recruited as a convenience sample)

**n= 516** participants

underwent the extended BP measurement protocol

**n= 511** participants

(187 men, 324 women)

**n= 20** participants

repeated measurements within 34 days for reliability assessment

n=5

excluded with

missing data on BP measurements

*BP: Blood pressure, BMI: body mass index, WC: waist circumference*

Text summary: Supplementary Table 1 shows the reliability of standing systolic blood pressure based on 28 and 20 participants in NAKO-pretest and MetScan studies, respectively

**Supplementary Table 1. Reliability of systolic blood pressure measurements in the NAKO-pretest and in the MetScan study.**

| **Systolic Blood pressure** | **n** | **Day** | **Mean ± SD** | **ICC** | **95% CI** |
| --- | --- | --- | --- | --- | --- |
| **NAKO-pretest study**  Mean days ±SD between first and second measurements = 10.2±3.9 days | | | | | |
| *Sitting 2_3* | 28 | 1 | 126.3±14.8 | 0.68 | 0.43-0.83 |
|  | 28 | 2 | 120.8±13.4 |  |  |
| *Standing 1* | 28 | 1 | 129.3±13.8 | 0.43 | 0.08-0.68 |
|  | 28 | 2 | 122.7±13.3 |  |  |
| *Standing 2* | 28 | 1 | 128.5±15.0 | 0.65 | 0.38-0.82 |
|  | 28 | 2 | 121.5±14.9 |  |  |
| *Standing 3* | 28 | 1 | 129.3±15.5 | 0.68 | 0.43-0.84 |
|  | 27 | 2 | 123.1±14.9 |  |  |
| *Standing 4* | 28 | 1 | 130.0±15.6 | 0.69 | 0.44-0.84 |
|  | 28 | 2 | 121.9±14.8 |  |  |
| *Standing 5* | 28 | 1 | 128.2±15.5 | 0.69 | 0.45-0.84 |
|  | 28 | 2 | 123.1±14.2 |  |  |
| *Standing 2_3_4* | 28 | 1 | 129.2±15.0 | 0.70 | 0.45-0.85 |
|  | 27 | 2 | 122.4±14.5 |  |  |
| **MetScan study**  Mean days ±SD between first and second measurements = 34.1±8.8 days | | | | | |
| *Sitting 2_3* | 20 | 1 | 120.3±16.7 | 0.81 | 0.59-0.92 |
|  | 20 | 2 | 116.4±12.8 |  |  |
| *Standing 1* | 20 | 1 | 127.9±16.2 | 0.72 | 0.44-0.88 |
|  | 20 | 2 | 120.2±15.7 |  |  |
| *Standing 2* | 20 | 1 | 123.2±16.1 | 0.84 | 0.65-0.93 |
|  | 20 | 2 | 116.9±14.9 |  |  |
| *Standing 3* | 20 | 1 | 122.1±18.6 | 0.87 | 0.71-0.94 |
|  | 20 | 2 | 118.5±15.9 |  |  |
| *Standing 4* | 20 | 1 | 121.6±16.4 | 0.82 | 0.61-0.92 |
|  | 20 | 2 | 117.7±14.8 |  |  |
| *Standing 2_3_4* | 20 | 1 | 122.3±16.7 | 0.86 | 0.70-0.94 |
|  | 20 | 2 | 117.7±14.9 |  |  |

*ICC: intraclass correlation coefficient., SD: standard deviation.CI: confidence intervals*

Text summary: Supplementary Table 2 shows that the distribution of characteristics of the study populations was, in general, similar for those whom did not have missing variables on covariates.

**Supplementary Table 2. Distribution of baseline characteristics of the study population without missing data in the covariates of the NAKO pretest and the MetScan**

|  | **NAKO-pretest study** | | |  | **MetScan study** | | |
| --- | --- | --- | --- | --- | --- | --- | --- |
|  | **All**  n=405 | **Men**  n=158 | **Women**  n=247 |  | **All**  n=501 | **Men**  n=183 | **Women**  n=318 |
| Age (years) | 53 (43-61) | 55 (44-62) | 52 (42-60) |  | 52 (36-64) | 55 (40-68) | 52 (34-62) |
| Monthly household income, % |  |  |  |  |  |  |  |
| <1500 € | 19.2 | 15.5 | 21.5 |  | 9.8 | 5.4 | 12.6 |
| 1500-2999 € | 40.7 | 42.6 | 39.5 |  | 44.5 | 38.7 | 48.0 |
| 3000-5999 € | 37.3 | 39.8 | 35.6 |  | 39.8 | 47.0 | 35.5 |
| ≥ 6000 € | 2.9 | 2.0 | 3.4 |  | 5.8 | 8.9 | 3.9 |
| Current smokers^a^, % | 23.9 | 31.8 | 18.9 |  | 9.8 | 8.7 | 6.6 |
| Diabetes Mellitus, % | 6.2 | 6.3 | 6.1 |  | 7.4 | 8.2 | 6.9 |
| Hypertension, % | 30.2 | 36.3 | 26.3 |  | 28.3 | 32.8 | 25.8 |
| Stroke, % | 2.7 | 3.8 | 2.0 |  | 1.6 | 0.5 | 2.2 |
| Previous MI, % | 1.2 | 3.2 | - |  | 1.4 | 1.6 | 1.3 |
| BMI, kg/m² | 25.5 (22.9-29.3) | 27.1 (24.6-29.7) | 24.4 (21.9-28.3) |  | 25.4 (22.5-28.3) | 25.9 (23.8-28.7) | 24.5 (21.7-28.3) |
| WC, cm | 89.5 (79.2-99.0) | 96.0 (90.3-105.5) | 83.8 (76.4-92.9) |  | 88.0 (77.9-99.0) | 95.3 (87.1-105.1) | 83.1 (74.0-93.2) |
| Waist-hip ratio | 0.9 (0.8-0.9) | 1.0 (0.9-1.0) | 0.8 (0.8-0.9) |  | 0.9 (0.8-0.9) | 0.9 (0.9-1.0) | 0.8 (0.7-0.9) |
| Waist-height ratio | 0.5 (0.5-0.6) | 0.5 (0.5-0.6) | 0.5 (0.5-0.6) |  | 0.5 (0.4-0.6) | 0.5 (0.5-0.6) | 0.5 (0.4-0.6) |
| Total cholesterol, mg/dL | 224.0  (189.5-247.5) | 222.2  (189.5-247.0) | 224.3  (189.5-247.0) |  | 216.6  (185.6-243.6) | 212.7  (185.6-239.8) | 216.6  (185.6-247.5) |
| HDL-cholesterol, mg/dL | 54.1 (46.4-69.6) | 46.4 (42.5-58.0) | 61.9 (50.3-73.5) |  | 58.0 (50.3-69.6) | 54.1 (46.4-58.0) | 61.9 (54.1-73.5) |
| LDL-cholesterol, mg/dL | - | - | - |  | 131.5 (108.3- 158.6) | 135.3 (108.3- 162.4) | 131.5 (108.3- 158.6) |
| HbA1c, mmol/mol | 37.5 (35.3-40.8) | 38.6 (36.4-40.8) | 37.5 (34.7-39.7) |  | 35.0 (32.0-38.0) | 35.0 (32.0-38.0) | 35.1 (32.0-38.6) |
| Antihypertensive therapy, % | 24.9 | 31.6 | 20.6 |  | 24.3 | 27.9 | 22.3 |
| SBP (mmHg) | 125 (115-135) | 129 (121-139) | 121 (111-133) |  | 121 (112-133) | 129 (119-138) | 116 (108-126) |
| DBP (mmHg) | 77 (71-84) | 80 (74-85) | 75 (69-83) |  | 76 (70-82) | 79 (73-84) | 74 (68-80) |
| Ambient temperature (°C) | 23.3 (22.7-24.1) | 23.3 (22.7-24.0) | 23.3 (22.6-24.1) |  | 22.0 (21.0-23.0) | 22.0 (21.0-23.0) | 22.0 (21.0-23.0) |
| Postural changes in SBP, mmHg | 1.7 (-2.7-5.8) | 1.7 (-3.0-5.7) | 1.7 (-2.0-6.0) |  | 2.0 (-1.8-6.0) | 1.8 (-2.5-5.8) | 2.0 (-1.5-6.2) |
| Postural change in SBP (in mmHg) category, % |  |  |  |  |  |  |  |
| Decline ≤-10 | 4.4 | 3.8 | 4.9 |  | 3.9 | 2.7 | 4.7 |
| Decline >-10 to ≤-5 | 11.4 | 11.4 | 11.3 |  | 9.6 | 9.8 | 9.4 |
| Decline >-5 to Increase ≤5 | 54.3 | 54.4 | 54.2 |  | 56.1 | 59.5 | 54.1 |
| Increase ≥5 to ≤10 | 20.9 | 23.4 | 19.4 |  | 21.4 | 20.8 | 21.7 |
| Increase >10 | 8.9 | 6.9 | 10.1 |  | 8.9 | 7.1 | 10.1 |

*Continuous variables expressed as median (IQR).*

*CVD: cardiovascular diseases, MI: myocardial infarction, BMI: body mass index, WC: Waist circumference SBP: systolic blood pressure, DBP: diastolic blood pressure, HbA1c: glycated hemoglobin.*

*^a^ Smoker variable: NAKO-pretest: never, current, former-smokers. MetScan: never, current, former-smokers defined as having smoked until 3 months previous to the enrollment.*

*Missing values: Monthly household income (n=24, n=54), current smokers (n=17 in NAKO-pretest), hypertension (n=1 in NAKO-pretest), previous MI (n=1), total cholesterol (n=2, n=11), HbA1c (n=98, n=13), HDL-cholesterol (n=106, n=11), LDL-cholesterol (n=11 in MetScan), antihypertensive therapy (n=7 in NAKO-pretest), in NAKO-pretest and MetScan, respectively.*

Text summary: Supplementary Table 3 shows the stratified analysis of the association between anthropometric measurements and postural blood pressure, according to values of sitting blood pressure.

**Supplementary Table 3. Association between anthropometric measurements and postural blood pressure by sitting blood pressure, expressed as β coefficient (β), 95% confidence intervals (CI).**

|  | **NAKO-pretest study** | | | | **MetScan** | | | |
| --- | --- | --- | --- | --- | --- | --- | --- | --- |
|  | **BP <140/90** | | **BP ≥140/90** | | **BP <140/90** | | **BP ≥140/90** | |
|  | **n** | **dSBP (95% CI), mmHg** | **n** | **dSBP (95% CI), mmHg** | **n** | **dSBP (95% CI), mmHg** | **n** | **dSBP (95% CI), mmHg** |
| ***BMI, per 5 kg/m^2^*** | | | | |  |  |  |  |
| Model 1 | 411 | 0.61 (-0.17-1.39) | 95 | 3.30 (1.92-4.68) | 425 | 0.90 (0.14-1.66) | 86 | 1.91 (0.28-3.54) |
| ***WC, per 5 cm*** | | | | |  |  |  |  |
| Model 1 | 411 | 0.08 (-0.22-0.38) | 95 | 1.37 (0.79-1.94) | 425 | 0.33 (0.04-0.61) | 86 | 0.85 (0.24-1.45) |
| Model 2 | 411 | -0.61 (-1.26-0.05) | 95 | 0.66 (-0.67-2.00) | 425 | 0.13 (-0.49-0.75) | 86 | 1.04 (-0.32-2.40) |

*BP: blood pressure (systolic blood pressure/diastolic blood pressure), BMI: body mass index, WC: Waist circumference*

*Model 1: adjusted by age, sex, diabetes, and stroke. Model 2: Model 1 additionally adjusted for BMI.*

Text summary: Supplementary Table 4 shows the distribution of baseline characteristics of participants in which the version 1 of the extended blood pressure measurement protocol was applied.

**Supplementary Table 4. Distribution of selected baseline characteristics of study participants with version 1 of the extended blood pressure measurement in the NAKO-pretest**

| **Participants with complete variables on BMI, WC and Δ SBP** | | n=117 |
| --- | --- | --- |
| Men/Women, n |  | 48/69 |
| Age (years), median (IQR) | | 49 (37-59) |
| Monthly household income, % | |  |
| <1500 € |  | 33.6 |
| 1500-2999 € |  | 31.8 |
| 3000-5999 € |  | 28.9 |
| ≥ 6000 € |  | 5.6 |
| Current smokers, % |  | 29.3 |
| Diabetes Mellitus, % |  | 5.1 |
| Hypertension, % |  | 23.1 |
| Stroke, % |  | 1.7 |
| Previous MI, % |  | 2.6 |
| BMI (kg/m²), median (IQR) | | 24.6 (22.3-28.6) |
| WC (cm), median (IQR) | | 89.3 (78.9-98.3) |
| Waist-hip ratio, median (IQR) | | 0.9 (0.8-0.9) |
| Waist-height ratio, median (IQR) | | 0.5 (0.5-0.6) |
| Total cholesterol (mg/dL*)*, median (IQR) | | 209 (181.7-236.0) |
| HDL-cholesterol (mg/dL*)*, median (IQR) | | 61.8 (50.3-79.2) |
| HbA1c (mmol/mol*)*, median (IQR) |  | 38 (35.8-40.2) |
| Antihypertensive therapy, % | | 24.7 |
| SBP (mmHg), median (IQR) | | 122 (115-131) |
| DBP (mmHg), median (IQR) | | 76 (70-82) |
| Ambient temperature (°C), median (IQR) | | 25.3 (24.6-26.1) |
| Postural changes in SBP (mmHg), median (IQR) | | 2.5 (-1.7-7.3) |
| Decline ≤ -10, % | | - |
| Decline >-10 to ≤-5, % | | 7.7 |
| Decline >-5 to Increase ≤ 5, % | | 54.7 |
| Increase ≥ 5 to ≤ 10, % | | 23.9 |
| Increase >10, % | | 13.7 |

*IQR: interquartile range, MI: myocardial infarction, BMI: body mass index, WC: Waist circumference, HbA1c: glycated hemoglobin, SBP: systolic blood pressure, DBP: diastolic blood pressure. Missing variables: HDL cholesterol=101, HbA1c n=101, current smokers n=5*

Text summary: Supplementary Table 5 shows the age-stratified association between anthropometric measurements and postural changes in systolic blood pressure.

**Supplementary Table 5.**

**Supplementary Table 5. Association between anthropometric measurements and postural changes in systolic blood pressure by age, expressed as β coefficient (β), 95% confidence intervals (CI).**

|  | **NAKO-pretest study** | | | | **MetScan** | | | |
| --- | --- | --- | --- | --- | --- | --- | --- | --- |
|  | **age <60** | | **age ≥60** | | **age <60** | | **age ≥60** | |
|  | **n** | **dSBP (95% CI), mmHg** | **n** | **dSBP (95% CI), mmHg** | **n** | **dSBP (95% CI), mmHg** | **n** | **dSBP (95% CI), mmHg** |
| ***BMI, per 5 kg/m^2^*** | | | | |  |  |  |  |
| Model 1 | 352 | 1.40 (0.59-2.22) | 154 | 1.73 (0.44-3.01) | 326 | 1.32 (0.56-2.01) | 185 | 1.61 (0.19-3.04) |
| ***WC, per 5 cm*** | | | | |  |  |  |  |
| Model 1 | 352 | 0.39 (0.08-0.71) | 154 | 0.59 (0.07-1.11) | 326 | 0.48 (0.18-0.77) | 185 | 0.65 (0.15-1.15) |
| Model 2 | 352 | -0.35 (-1.00-0.31) | 154 | -0.13 (-1.31-1.04) | 326 | 0.13 (-0.53-0.79) | 185 | 0.64 (-0.39-1.67) |

*BMI: body mass index, WC: Waist circumference, SBP: systolic blood pressure*

*Model 1: adjusted by sex, seated SBP, diabetes, and stroke. Model 2: Model 1 additionally adjusted for BMI.*

*Interaction terms: p-value for BMI x age60 was 0.22, p-value for WC x age60 was 0.23 in the NAKO-pretest. In the MetScan study, p-value for BMI x age60 was 0.50 and p-value for WC x age60 was 0.32.*
